# Triclabendazole and Other Fasciolicides: Resistance of *Fasciola hepatica* in Ruminants

**DOI:** 10.3390/ani16071044

**Published:** 2026-03-29

**Authors:** Meiru Hou, Junfeng Gao, Xuewei Liu, Jiawang Zhou, Tianshuai Ma, Ying Zhang, Hongyu Qiu, Chunren Wang

**Affiliations:** 1 Key Laboratory of Prevention and Control of Zoonotic Diseases of Daqing, College of Animal Science and Veterinary Medicine, Heilongjiang Bayi Agricultural University, Daqing 163319, China; houmeiru168@126.com (M.H.); gaojunfeng_2005@163.com (J.G.); 19845919553@163.com (X.L.); 18378465906@163.com (J.Z.); 13136815086@139.com (T.M.); qiuhongyu95@163.com (H.Q.); 2 College of Food Science, Heilongjiang Bayi Agricultural University, Daqing 163319, China; 3 College of Agriculture, Jinhua University of Vocational Technology, Jinhua 321000, China; yingzhang8609@sina.com

**Keywords:** *Fasciola hepatica*, fasciolicides, anthelmintics, resistance, ruminants

## Abstract

Fasciolosis is a globally prevalent trematode infection of major veterinary and public-health relevance. Juveniles migrate through liver tissue for weeks before patency, so clinically important infection may occur while faecal egg output is undetectable, complicating control and interpretation of apparent treatment failure. Triclabendazole (TCBZ) remains central because it targets both immature and adult flukes, but sustained use has been accompanied by geographically expanding reports of reduced efficacy and confirmed resistance. Most alternative fasciolicides, such as albendazole, closantel, oxyclozanide, rafoxanide, clorsulon and nitroxynil, are largely adulticidal and used alone or in combinations, yet reports of reduced efficacy/resistance are increasing worldwide. This review summarises drugs in current use and reported resistance status, and outlines a practical pathway for detecting and confirming resistance. We then appraise leading mechanistic hypotheses for TCBZ resistance as a central case study, organised around microtubule-associated phenotypes, reduced effective drug exposure, genetic architecture with tissue context, stress response and detoxification capacity, and we highlight mechanistic gaps for other fasciolicides. Finally, we discuss management implications, including monitoring-guided stewardship, stage-appropriate drug selection, rational combinations, integrated parasite management, and identify near-term priorities for harmonised surveillance, improved diagnostics and tool development. This review updates the resistance landscape and supports practical, monitoring-guided control of fasciolosis.

## Simple Summary

Fasciolosis is a common parasite infection of cattle and sheep that often leads to livers being rejected at slaughter. Control is difficult because young flukes can damage the liver for weeks before eggs appear in faeces, so infections may be important but hard to detect early and apparent treatment failure can be difficult to interpret. Triclabendazole is widely used because it is active against both immature and adult flukes, but reduced efficacy and drug resistance are increasingly reported. Most alternative fasciolicides are largely adulticidal and used as Triclabendazole alternatives, yet emerging resistance has also been reported. This review summarises drugs in current use, where resistance has been reported, and practical steps to investigate suspected resistance using complementary tests. It also outlines the main biological explanations for reduced drug susceptibility and highlights priorities for better monitoring, improved diagnostics and future tool development to support sustainable fasciolosis control.

## 1. Introduction

Fasciolosis is a globally prevalent trematode infection of major veterinary and public-health relevance. In endemic areas, *Fasciola hepatica* is widespread in cattle and sheep and can reach sufficiently high prevalence to cause clinically significant disease. Beyond overt outbreaks, infection imposes substantial but often underestimated chronic production losses through reduced weight gain, milk yield, fertility and carcass value, while also increasing susceptibility to secondary bacterial infections and leading to frequent liver condemnation at slaughter.

A defining biological feature that shapes both control and interpretation of treatment failure is the pronounced stage structure of infection. Following ingestion of metacercariae, newly excysted juveniles penetrate the intestinal wall, traverse the peritoneum and migrate through hepatic parenchyma before reaching the bile ducts over several weeks. In general, excysted parasites typically establish in the bile ducts by around 6–8 weeks post infection, whereas eggs usually begin to appear in faeces only from approximately 8–12 weeks post infection. Because pathology, diagnostic sensitivity and drug efficacy differ sharply between immature and adult stages [1], early infections can be clinically consequential yet diagnostically silent—damage is accruing while eggs remain undetectable—creating a persistent diagnostic and therapeutic gap in routine field practice. This stage-dependent biology also makes treatment timing especially important, because apparent drug failure may reflect mismatch between parasite stage structure and drug activity rather than true resistance.

Triclabendazole (TCBZ) has therefore remained central to fasciolosis control because it is highly active against both immature and adult flukes [2]. However, sustained reliance—combined with imperfect dosing, operational errors, and high reinfection pressure—has driven selection and the spread of reduced efficacy and confirmed resistance since the mid-1990s [3,4]. Other fasciolicides provide partial alternatives but typically exhibit narrower stage activity, often limited to adult flukes, complicating both control decisions and failure attribution.

In this review, we focus on *F*. *hepatica* in cattle and sheep and provide an integrated overview spanning drug use, resistance emergence, diagnostic workflows and mechanistic models. We summarise fasciolicides in current use and the reported resistance status, including recently reported field evidence, outline practical approaches for detecting and confirming resistance, and review leading mechanistic hypotheses with TCBZ as the central case study, thereby helping to fill the gap between recent descriptive reviews and the need for more practical guidance on resistance management. We then discuss alternative control options and their implications for management, with a particular emphasis on the practical integration of these components within a closed-loop approach to resistance detection, confirmation and follow-up surveillance, and conclude by highlighting priority research gaps and near-term directions for surveillance, diagnostics and tool development.

## 2. Search Strategy and Selection Criteria

This review was prepared as a structured narrative review of fasciolicide resistance in *F. hepatica* infecting ruminants. Literature was searched in PubMed, Web of Science, Scopus, and Google Scholar up to 12 December 2025 using combinations of terms related to *F. hepatica*, fasciolicides, anthelmintic resistance, reduced efficacy, diagnosis, and integrated parasite management. Additional relevant references were identified through citation tracking of key review and original research papers. Original studies, field reports, diagnostic studies, and selected surveillance reports relevant to fasciolicide efficacy or resistance in cattle and sheep were included. Studies focused exclusively on human fascioliasis, unrelated parasites, or articles lacking sufficient relevance or methodological detail were excluded. The retrieved literature was synthesised thematically to support the sections on drug use and resistance status, diagnostic approaches, mechanistic evidence, and management implications.

## 3. Fasciolicides in Use and Resistance Status

Among available fasciolicides, TCBZ is widely used because it is highly active against both immature and adult flukes. Since its introduction for use in livestock in 1983, resistance has progressively emerged as a slow-moving, globally disseminating phenomenon closely linked to production practices. In contrast, most other fasciolicides, such as albendazole (ABZ), rafoxanide, clorsulon and closantel, are typically stage-restricted and therefore deployed primarily as adult-stage options or as components of combination strategies. Nevertheless, reports consistent with reduced efficacy or resistance have also accumulated across multiple regions worldwide (Table 1).

TCBZ is unique in its activity against both immature and adult flukes. Its use in livestock has been reported since 1983 [5], Reports of reduced efficacy in *F. hepatica* have since accumulated as a gradual global emergence, best interpreted as a geographically expanding, production-linked phenotype rather than a set of isolated treatment failures [6]. Recent genomic studies further suggest that TCBZ resistance in *F. hepatica* is not explained by a single universal mechanism, but rather by a geographically heterogeneous genetic architecture. A major resistance locus with dominant inheritance has been identified in UK material, whereas population genomic data from Peru support independent and non-parallel resistance signatures across field populations [7,8]. Early signals were noted in Australia in the mid-1990s, followed soon after by reduced field efficacy reported in Scottish sheep in the UK [9]. By the turn of the millennium, compelling farm-level evidence accumulated across north-western Europe, including strikingly poor post-treatment egg count reductions in mixed sheep–cattle systems in the Netherlands and reports from Wales, followed by experimental confirmation using a resistant isolate [10,11,12]. Subsequent reports from Spain and Ireland indicated that reduced TCBZ efficacy was not confined to a single management context, and UK case investigations increasingly framed resistance as an economic and welfare issue [13,14,15]. Beyond Europe, expanding reports documented reduced efficacy in South America, first in Brazil, Argentina and later in Peru and Chile, with in vivo confirmation supporting causal attribution [16,17,18,19]. Composite faecal egg count reduction test (FECRT) approaches and other methodological advances enabled broader surveillance, helping the field shift from isolated suspicion to large-scale confirmation [20,21,22]. In Australia, resistance became firmly established in cattle, supported by coproantigen- and egg-count-based reductions and, critically, recovery of live flukes after dosing, with subsequent regional studies underscoring a substantial endemic burden [23,24,25]. Recent work highlights pronounced within-country heterogeneity in susceptibility, while new national first reports from New Zealand and therapeutic failure signals in Egypt suggest that the map of TCBZ resistance continues to expand [26,27,28].

Beyond TCBZ, most alternative fasciolicides are stage-restricted and are often deployed as adult-stage options or as components of combination strategies. ABZ was first reported for use in livestock in 1976 [29], and the first clear field signal in sheep came from north-west Spain, where reduced ABZ efficacy was documented alongside reduced TCBZ efficacy, foreshadowing that benzimidazole resistance could co-emerge within intensively treated fluke populations [13]. A second focal node appeared in South America, where a defined Argentine field isolate maintained under laboratory conditions showed markedly reduced ABZ flukicidal efficacy despite adequate systemic exposure to active metabolites, supporting true parasite resistance rather than product quality or underdosing [30]. More recent field evidence also indicates that benzimidazole resistance in *F. hepatica* may be broader and more heterogeneous than previously recognised. In naturally infected sheep, albendazole resistance and reduced efficacy of other benzimidazole formulations were reported, supporting continued consideration of β-tubulin-related biology while arguing against a simple class-wide target-site model [31].

Rafoxanide was first described for the control of ruminant flukes in 1970 [32]. Earlier field data from Turkey had already suggested reduced efficacy of rafoxanide, together with albendazole, in naturally infected cattle, indicating that drug-specific signals extended beyond a single setting [33]. Extending the geography and drug classes implicated, a randomized field trial in naturally infected cattle in Beni-Suef, Egypt, found persistently suboptimal faecal egg count reductions with ABZ and rafoxanide, whereas oxyclozanide and TCBZ achieved complete suppression by day 14, consistent with ABZ and rafoxanide resistance and providing the first field signal for reduced rafoxanide efficacy in Egypt [34].

The efficacy of clorsulon in sheep and cattle was reported in 1977 [35]. Subsequent work in Spain extended the phenotype to multi-drug contexts, where a flock in León province showed resistance to ABZ and clorsulon, and while either drug alone performed poorly, co-administration at full recommended doses restored high efficacy, suggesting that combination regimens may partially compensate for single-agent failure in some settings [36]. Clorsulon has long been positioned as an alternative option against adult flukes when TCBZ efficacy is compromised, and early experimental evidence showed that clorsulon retained activity against adult TCBZ-resistant (TCBZ-R) *F. hepatica* [37]. Nonetheless, resistance-associated reductions in clorsulon efficacy have been reported, most notably in Spanish sheep flocks with concurrent ABZ resistance, where clorsulon performance against adult and immature stages fell below expected benchmarks [2].

Closantel targets adults and late immature stages, and it was described as a ruminant fasciolicide in 1977 [38]. The first well-documented closantel failure against *F. hepatica* in cattle was reported in south-west Sweden, where lack of reduction in faecal egg counts (FEC) and persistent coproantigen positivity after pour-on administration could not be explained by immature-stage survival, raising concern for emerging resistance and formulation-dependent underexposure [39].

Oxyclozanide, which is mainly adulticidal, was first reported for the treatment of fasciolosis in sheep and cattle in 1966 [40]. Nitroxynil was first described as an effective adulticidal treatment for fasciolosis in 1969 [41]. Evidence for resistance remains limited and has not been independently confirmed for both fasciolicides.

**Figure 1:**
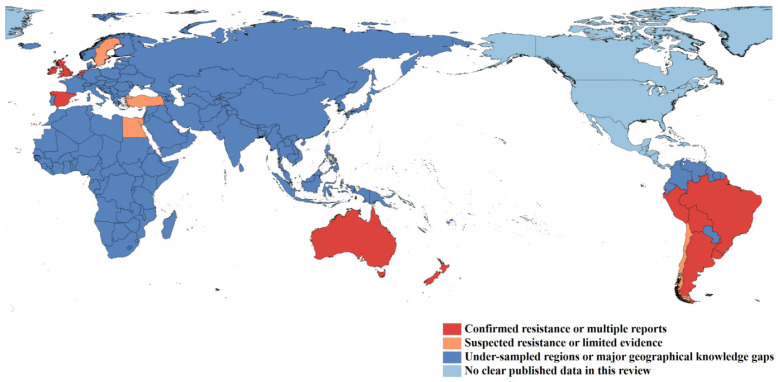
Global distribution of published reports of fasciolicide resistance and major geographical knowledge gaps in *Fasciola hepatica*. Note: this map reflects the current geographical distribution of published evidence rather than the true absence of resistance in regions with limited surveillance or reporting.

Overall, these reports show that fasciolicide resistance and reduced efficacy have been documented across multiple regions, but the evidence remains concentrated in a limited number of countries. Large parts of Asia, Africa, and other regions remain under-sampled or lack clear published field data (Figure 1). Therefore, the current global picture should be interpreted as a reflection of uneven surveillance and reporting rather than the true absence of resistance.

## 4. Method of Detecting and Confirming Resistance

Robust diagnosis of fasciolicide resistance is hindered by the absence of widely adopted, fit-for-purpose field standards. The FECRT remains the most commonly used approach for detecting TCBZ resistance, but it has well-recognized limitations, particularly when baseline egg output is low, aggregation is high, or stage composition is shifting. Importantly, FEC-based readouts cannot, by themselves, distinguish true resistance from reinfection, stage mismatch, or operational failures. Recent field evidence from Australia further highlights that applying existing W.A.A.V.P. style criteria to fasciolicide resistance can be challenging under real-world conditions, supporting the need for Fasciola-specific interpretive guidance and multimodal confirmation pathways [42]. As a result, suspicion of reduced efficacy should progress through a confirmation pathway, using complementary tests whenever feasible (Table 2).

### 4.1. Faecal Egg Count Reduction Test

FECRT is inexpensive and field-feasible, inferring efficacy by comparing pre- and post-treatment FEC. However, diagnostic reliability depends on baseline egg output, aggregation of parasites, and the threshold chosen [43]. It is particularly prone to variability when infection intensity is low and is influenced by sampling error, egg-shedding dynamics, and stage composition [2]. These sources of uncertainty can inflate both false resistance signals and false reassurance, especially when animals are sampled outside optimal post-treatment windows. Thus, while FECRT is useful for early detection, it is best treated as a screening step and should be paired with an independent indicator of active infection and clearance.

Recent advancements have improved FEC methods for point-of-care or out-of-laboratory settings. Automated systems such as FECPAKG2 (Techion, Dunedin, NZ) and ParaSight System (ParaSight Systems Inc., Lexington, KY, USA) have been adopted for field use [44]. FECPAKG2 allows testing on-farm or in the laboratory, with a sensitivity of 35 epg for sheep and 20 epg for cattle. It is suitable for routine monitoring but typically requires appropriately designed pooling and replication when used for anthelmintic resistance testing [45]. Similarly, the ParaSight System uses electronic visualization and fluorescence analysis to detect labelled helminth eggs, offering a sensitivity range of 1–6 epg [46]. Despite clear technical promise, cost per individual sample and limited validation specifically for resistance workflows remain barriers to broad adoption.

In contrast, FLOTAC provides a more sensitive and accurate copromicroscopic method, with high egg recovery and lower variability than traditional techniques [47,48]. For *F. hepatica*, a modified zinc sulphate FLOTAC system has been used successfully in large-scale on-farm surveys and for drug efficacy testing [44,49]. It is well-suited to pooled testing and offers a low-cost alternative for drench-resistance investigations [50,51,52].

### 4.2. Coproantigen Reduction Testing (CRT)

Coproantigen ELISA (cELISA) detects Fasciola antigens in faeces and therefore provides evidence of active infection, making CRT a practical tool to assess treatment response when egg counts are low, variable, or prepatent. The most widely used platform is the monoclonal antibody MM3-based assay, which was later commercialised as the BIO K201 ELISA kit and has been used in numerous studies since 2007 [53]. The target antigen is likely a cathepsin-type enzyme, as immunolabelling is restricted to gastrodermal cells [54,55]. The assay is highly specific for *F. hepatica*, with no reported cross-reactivity to selected trematodes, cestodes, gastrointestinal nematodes, or coccidia in validation studies [55,56], and is sensitive enough to detect infections of as few as one fluke in sheep and cattle [57]. Because coproantigens persist only for the duration of infection, they indicate current infection rather than historical exposure. Coproantigens can typically be detected from 5 to 6 weeks post infection, broadly corresponding to bile-duct entry [58], providing a window that is often earlier than patency-based egg detection.

CRT has increasingly been used not only for diagnosis but also for determining drug efficacy and, by extension, supporting resistance investigations. Experimental infections with TCBZ-susceptible and -resistant isolates showed that CRT tracks true efficacy confirmed by necropsy and can discriminate susceptible versus resistant outcomes, supporting its role as an independent confirmation step alongside FECRT [59]. Field studies further underscore CRT’s operational value. Under farm conditions, CRT produced group-level efficacy estimates comparable to FECRT at 7–21 days post treatment and helped document TCBZ treatment failure signals despite accurate dosing, although individual-level discordance between tests can occur [60]. In Sweden, CRT detected complete ABZ treatment failure while TCBZ rapidly cleared coproantigen and eggs within a week, supporting utility beyond TCBZ [61]. In Australia, coproantigen ELISA enabled efficient pooled screening with strong agreement between bulk and individual testing, and CRT supported confirmation of TCBZ resistance while indicating that adulticidal alternatives could still remove adult stages [24]. More recently, a German multi-farm survey reported that low egg shedding often limited FECRT feasibility, so CRT was implemented in parallel and helped identify a flock with poor TCBZ response, illustrating how CRT can preserve diagnostic resolution when egg-based readouts are constrained [62].

### 4.3. Controlled Efficacy Test (CET)

The CET is the most definitive approach for confirming fasciolicide resistance because it directly quantifies fluke survival after treatment through necropsy, enabling stage stratification and avoiding the confounding effects of egg-shedding dynamics. CET therefore provides the most robust evidence of true drug efficacy and is particularly valuable for resolving contested reports and validating field-based proxies such as FECRT and CRT [63]. However, CET is resource-intensive, requires specialised facilities and ethical approval, and is rarely feasible for routine on-farm surveillance. As a result, CET is best positioned as a reference confirmation tool for sentinel investigations and for establishing well-characterised susceptible and resistant isolates for downstream mechanistic and molecular studies.

### 4.4. Egg Development and Hatching Test (EDHT)

Because slaughtering livestock specifically to assess efficacy against *F. hepatica* is impractical in most production settings, in vitro phenotypic assays have been developed to provide rapid readouts for resistance evaluation. The EDHT quantifies the ability of *F. hepatica* eggs to develop and hatch following in vitro exposure to a drug, thereby assessing ovicidal or embryostatic activity [64,65]. A practical advantage is that eggs derived from a single animal can be used to probe the susceptibility of the local parasite population to one or more compounds, making EDHT a comparatively accessible and cost-effective complement to field tests. Agreement between in vitro and in vivo outcomes has been reported for ABZ resistance, supporting EDHT as a plausible phenotyping option where CET is not feasible [66].

However, EDHT performance remains limited by methodological heterogeneity. Protocols vary substantially in egg source, incubation conditions, exposure duration, drug concentration ranges, and whether parent compounds or metabolites are used, including differences in solvent systems [67]. This between-study variability reduces comparability and complicates threshold-setting, underscoring the need for agreed minimal standards and reporting. Despite these constraints, EDHTs are particularly suited to evaluating benzimidazole-class compounds, where they can discriminate susceptible versus resistant isolates under controlled conditions [66,68]. In addition, a recent modified egg hatch test study extended this approach to nitroxynil and albendazole sulfoxide, reporting concentration-dependent inhibition of egg development and hatch, with IC_50_ values of 0.043 μmol L^−1^ for nitroxynil and 0.00099 μmol L^−1^ for albendazole sulfoxide [69]. These findings support the potential to broaden egg-based in vitro phenotyping to additional fasciolicides and provide a basis for future development of field-applicable discriminatory thresholds. With protocol harmonisation, EDHT could provide a scalable laboratory adjunct for resistance monitoring and for mechanistic studies linking phenotype to exposure–response relationships.

### 4.5. Serology

Serological assays, including antibody-based ELISAs, are useful for exposure mapping and risk stratification at the herd level. A proteomics-guided study identified excretory/secretory biomarkers associated with in vitro TCBZ-sulfoxide outcomes and evaluated recombinant calreticulin and triose phosphate isomerase as antigens to probe molecular phenotypes in experimentally infected sheep. Sera from sheep infected with TCBZ-susceptible versus TCBZ-R isolates showed differential immunoreactivity to these antigens, suggesting potential utility as exploratory adjunct markers to support TCBZ efficacy assessment, independent of fully characterised resistance mechanisms [70]. However, antibody levels may remain elevated long after treatment, indicating past exposure rather than active infection. Overall, serological tests are therefore most useful for surveillance rather than post-treatment confirmation of reduced efficacy or resistance. Decisions about treatment efficacy should rely on more immediate indicators of infection, such as CRT and appropriately timed egg count reduction tests, rather than antibody persistence signals.

### 4.6. Molecular Diagnostics and Emerging Tools

Unlike gastrointestinal nematodes, where validated resistance loci enable marker-based surveillance, *F. hepatica* does not yet have universally accepted molecular markers for routine diagnosis of TCBZ resistance, although recent genome-wide work has identified a major locus associated with TCBZ resistance and shown dominant inheritance of this trait [7]. Molecular assays therefore primarily support infection quantification and provide a foundation for future resistance genotyping rather than currently enabling routine molecular diagnosis of TCBZ resistance.

#### 4.6.1. PCR and qPCR

Early work demonstrated that PCR could detect infection during the prepatent period and could be deployed alongside FECRT and CRT in field resistance investigations [71]. However, field comparisons highlighted that PCR sensitivity can be suboptimal if sample volume, egg concentration, and DNA extraction are not optimised [72]. To address these bottlenecks, a pelleting-based workflow was developed to concentrate eggs, followed by bead-beating and qPCR, enabling higher throughput, species differentiation between *F. hepatica* and *F. gigantica*, strong correlation with FEC, and robust performance even after storage in 70% ethanol [73]. Importantly, experimental data indicate that qPCR positivity closely tracks faecal egg appearance, limiting very early detection despite larger starting volumes [58]. This reinforces that molecular positivity does not resolve stage composition, and results can still be confounded by prepatency and reinfection dynamics.

A SYBR Green qPCR targeting ITS-2 has also been reported for sensitive laboratory diagnosis in sheep [74]. Beyond Fasciola, molecular identification is valuable in trematode-coendemic settings where egg morphology overlaps and co-infections can bias prevalence and egg-shedding patterns [75]. For resistance investigations, these tools are best viewed as complements that improve diagnostic resolution when egg counts are low, rather than replacements for phenotypic confirmation.

#### 4.6.2. LAMP and RPA

Isothermal amplification formats are increasingly enabling near-field testing. LAMP assays can achieve very high analytical sensitivity—down to a single spiked *F. hepatica* egg in faeces using nuclear targets—with no cross-amplification reported for selected helminths [76]. In cattle, integrating bead-beating into DNA extraction improved LAMP performance, with discrepancies versus conventional FEC in field comparisons likely reflect, at least in part, FEC false negatives at low burdens [77]. RPA provides another field-adaptable platform and has outperformed real-time PCR for detecting *F. hepatica* in low egg burden human stool, while maintaining 100% specificity. Furthermore, employing a lateral-flow readout enhanced its sensitivity even further [78].

Critically, although these molecular and isothermal assays can improve detection logistics, they currently do not diagnose resistance in the absence of validated resistance loci. Their near-term value for resistance management is therefore operational. They enable earlier or more reliable detection of active infection, supporting targeted treatment decisions, and strengthening interpretation of FECRT results through triangulation.

## 5. Mechanistic Hypotheses and Evidence Strength

### 5.1. Triclabendazole

#### 5.1.1. Microtubule-Associated Biology

Microtubule disruption is a consistent pharmacological signature of TCBZ action in *F. hepatica*, supported by extensive ultrastructural and immunolabelling evidence. Exposure to TCBZ and its metabolites disrupts secretory vesicle trafficking and cell division, accompanied by loss of tubulin immunostaining. Notably, these characteristic phenotypes are diminished or absent in TCBZ-R flukes, supporting the view that altered tubulin binding or microtubule responsiveness may represent an early working hypothesis [79].

However, sequencing and binding-site comparisons have not produced a simple, transferable β-tubulin resistance marker analogous to gastrointestinal nematodes. *F. hepatica* β-tubulin differs from mammalian tubulin at multiple residues, and Tyr200—classically implicated in benzimidazole resistance in nematodes—appears present even in susceptible *F. hepatica* isolates, undermining a straightforward codon 200 narrative [80,81]. Comparative sequencing has not identified consistent β-tubulin differences between TCBZ-susceptible (TCBZ-S) and TCBZ-R isolates that could explain resistance alone [81,82]. Likewise, β-tubulin isotypes show stage-specific expression, yet qPCR detected no significant transcriptional differences between susceptible Leon and resistant Oberon isolates. Only one homozygous change in β-tub4 was found, and modelling predicted minimal structural impact and limited relevance to proposed binding residues, arguing against β-tubulin mutation or altered expression as a primary driver [83].

Transcriptomic evidence nevertheless supports that microtubule-associated pathways can be remodelled in resistant phenotypes. In Latin American isolates with contrasting drug phenotypes, a TCBZ/ABZ–resistant isolate showed broad downregulation of cytoskeletal and microtubule machinery, including α/β-tubulins, kinesins and dyneins, consistent with altered microtubule-related biology as a resistance correlate rather than a single target mutation [84]. Collectively, these data support microtubule disruption as a robust phenotypic signature of TCBZ resistance, while weakening the hypothesis that TCBZ-R is explained by one universal β-tubulin substitution.

#### 5.1.2. Reduced Effective Drug Exposure

Across isolates, some of the most persuasive mechanistic evidence for reduced TCBZ susceptibility converges on a shared phenotype. This phenotype is lower effective intracellular exposure to the active drug, achieved through altered transport, altered biotransformation and drug sequestration. Recent genome-wide mapping has strengthened this interpretation by identifying a major resistance locus associated with TCBZ resistance, indicating that reduced drug exposure should now be considered in the context of a broader genomic region rather than only through earlier candidate gene hypotheses [7].

Transport and efflux mechanisms. Pharmacology-driven uptake/efflux studies indicate that TCBZ-R flukes can exhibit reduced intracellular accumulation of TCBZ and its active sulfoxide metabolite, and that efflux pump modulators can partially reverse key phenotypes [85]. In particular, the P-glycoprotein inhibitor R(+)-verapamil increased uptake/retention of TCBZ in TCBZ-R flukes and was associated with markedly increased tegumental disruption in vitro, supporting transporter-mediated efflux as a functional contributor to resistance in at least some isolates [86,87]. However, candidate SNP associations in transporter genes have shown weak reproducibility across populations, limiting their utility as universal diagnostics [88,89,90]. This is consistent with more recent evidence that resistance may reflect variation within a broader genomic locus rather than a single universally informative transporter marker [7]. In addition, although some microdissection-based approaches have yielded inconclusive results, more recent evidence summarised by Rufino-Moya et al. (2024) indicates that Pgp/ABC transporters have been identified in the tegument, gastrodermis, and reproductive organs of *F. hepatica*, supporting their anatomical distribution in tissues relevant to drug uptake, processing, and reproductive biology [91]. Taken together, current evidence supports reduced effective drug exposure as a persuasive mechanistic theme, but the causal molecular basis is not yet resolved to one single transporter gene or pathway.

Metabolic reprogramming and inhibitor rescue evidence. A second experimentally supported route to reduced effective exposure is altered metabolism of TCBZ and its metabolites. Ex vivo SEM-based phenotyping showed that TCBZ or TCBZ-sulfoxide alone caused less tegumental disruption in the resistant Oberon isolate than in susceptible Cullompton. Importantly, inhibiting parasite oxidative metabolism with methimazole markedly increased drug-associated surface damage in Oberon, with minimal enhancement in the susceptible isolate—consistent with resistance being partly mediated by altered biotransformation of TCBZ metabolites [92]. Parallel work showed that inhibiting cytochrome P450–mediated metabolism with piperonyl butoxide substantially increased TCBZ/TCBZ-sulfoxide–associated injury in the resistant isolate, further supporting a metabolism-linked component to reduced susceptibility [93]. Broader metabolite profiling has suggested that both TCBZ to TCBZ sulfoxide (TCBZ-SO) and TCBZ-SO to TCBZ sulfone (TCBZ-SO_2_) steps can be increased in some resistant backgrounds, implying faster conversion and a shifted balance among active versus less active metabolites [4]. Inhibitor profiling has also suggested Flavin-containing monooxygenase (FMO) pathways may dominate TCBZ metabolism in flukes, with stronger inhibitory effects of methimazole on sulfoxidation in TCBZ-R compared with TCBZ-S isolates, while cytochrome P450 inhibition showed smaller and more similar effects across isolates [67]. Beyond biochemical inference, independent studies have reported potentiation of TCBZ effects against resistant flukes by metabolic inhibitors, supporting causal plausibility through phenotype rescue [94].

Drug sequestration and reduced free-drug availability. Proteomics-based work supports a ligandin-like sequestration mechanism that may reduce free active drug. Comparative proteomics of susceptible Cullompton and resistant Sligo flukes exposed to TCBZ-SO showed higher basal Hsp90/Hsp70 in resistant parasites and distinct drug-responsive changes, including strong upregulation of fatty acid–binding protein. Binding assays confirmed direct interaction of Fh15 with TCBZ-SO, supporting the model that ligandin binding reduces free drug availability [95]. This interpretation is further strengthened by recent genome-wide mapping, which identified a major TCBZ-R locus containing a candidate fatty acid-binding protein (FABP) gene, consistent with a role for drug sequestration in limiting access of the compound to its microtubule target in the parasite [7]. In addition, extracellular vesicles have been proposed as a further non–target-site contributor to reduced exposure. In vitro exposure of adult flukes to TCBZ and its metabolites increased EV release substantially, and mass spectrometry detected TCBZ and TCBZ-SO within purified extracellular vesicles, supporting a model in which extracellular vesicles sequester and export drug-related compounds, lowering effective intracellular exposure under drug pressure [96].

Taken together, transporter modulation, metabolic inhibitor rescue, sequestration and EV export represent different angles on a common endpoint—reduced effective drug exposure—and are among the most compelling explanations because they link mechanistic perturbation to phenotypic change.

#### 5.1.3. Genetic Architecture and Tissue Context

Recent genomic and transcriptomic analyses have nominated multiple loci and pathways associated with TCBZ-R, including candidates linked to EGFR/PI3K/AKT–mTOR–S6K signalling and microtubule-related functions, with reported expression differences between TCBZ-S and TCBZ-R flukes [8]. Separately, resistance has been proposed to map to a dominant allele at a major genetic locus, containing genes related to membrane transport and signal transduction, with stage-dependent expression patterns strongest in adults [7]. These studies support a model in which resistance is genetically tractable but potentially polygenic and background-dependent, consistent with variable portability of single candidate markers across regions.

Spatial transcriptomics adds an important biological filter by placing candidate resistance genes into tissue context. A 2D atlas resolved tissue compartmentalization of detoxification families, with some ABC-B candidates enriched in reproductive tissues, whereas a tegument-enriched ABC transporter may be more plausible given tegumental drug uptake. This reinforces that resistance inference should prioritize genes expressed at drug-exposed interfaces and consider tissue-specific expression when selecting candidates for functional validation [97].

#### 5.1.4. Stress Response and Detoxification Capacity

Proteomics and biochemical studies suggest that downstream stress response capacity may modulate survival under drug pressure. Resistant flukes have been reported to show higher basal Hsp90/Hsp70 and distinct inducible stress signatures upon TCBZ-SO exposure, consistent with enhanced cellular stress management [4,95]. Glutathione S-transferase (GST) focused assays have also been developed to interrogate phase II detoxification systems as contributors to metabolism and resistance-associated phenotypes [98]. While these findings provide biologically coherent support for a tolerance network, they are frequently best interpreted as supportive or modulatory evidence—capturing what changes during drug challenge and survival—rather than proving a single initiating cause of resistance.

### 5.2. Mechanisms for Other Fasciolicides

Albendazole: Across two decades of work, β-tubulin has been examined in *F. hepatica* as the most plausible benzimidazole target, yet the data collectively argue against a simple single-mutation resistance model. The first characterization of an *F. hepatica* β-tubulin gene established baseline sequence features and highlighted multiple amino acid differences from nematode and cestode tubulins that may help explain the relatively limited fasciolicidal activity of most BZMs and the difficulty of extrapolating canonical nematode resistance rules to trematodes [98]. Building on this, structural modelling proposed that an inter-domain movement could transiently expose otherwise buried residues, including the region around codon 200, offering a mechanistic rationale for how BZMs might access the binding pocket and how codon-200 substitutions confer resistance in nematodes [99]. Subsequent transcriptomic surveys showed that adult *F. hepatica* expresses a complex tubulin repertoire with variation at key BZ-site residues, including different amino acids at β-tubulin position 200. However, these findings suggest that while β-tubulin remains the most plausible benzimidazole target in *F. hepatica*, current evidence does not support a simple target-site explanation for reduced susceptibility.

Salicylanilides (closantel, rafoxanide, oxyclozanide): Salicylanilides resistance mechanisms are understudied, and even their mode of action can be complex. For salicylanilides, there is relatively compelling evidence consistent with uncoupling of oxidative phosphorylation in flukes, supported by characteristic metabolic changes observed in vivo and in vitro [100,101]. Notably, for oxyclozanide, the absence of ATP depletion in vitro led to the hypothesis that the primary action might instead be neurotoxic [102].

Nitroxynil: The pharmacology underlying nitroxynil resistance remains understudied. Nitroxynil is traditionally classified as an uncoupler of oxidative phosphorylation. However, direct evidence in fluke-relevant experimental systems is limited, and many studies rely on supraphysiological concentrations. This interpretation is further weakened by the observation that oxidative phosphorylation accounts for only a minor fraction of *F. hepatica* energy metabolism [103]. Moreover, phenols can induce rapid spastic paralysis at concentrations comparable to therapeutically relevant blood levels, suggesting that altered membrane ion permeability may also contribute to the predominant phenotype [2].

Overall, the available evidence indicates that fasciolicide resistance in *F. hepatica* is mechanistically diverse and unevenly resolved across drug classes (Table 3). For TCBZ, reduced effective drug exposure currently has the strongest support, whereas for most alternative fasciolicides the mechanistic basis of reduced efficacy remains incompletely defined. In addition, no routinely validated molecular marker is yet available for field diagnosis across the major fasciolicides.

## 6. Management Protocol

**Figure 2:**
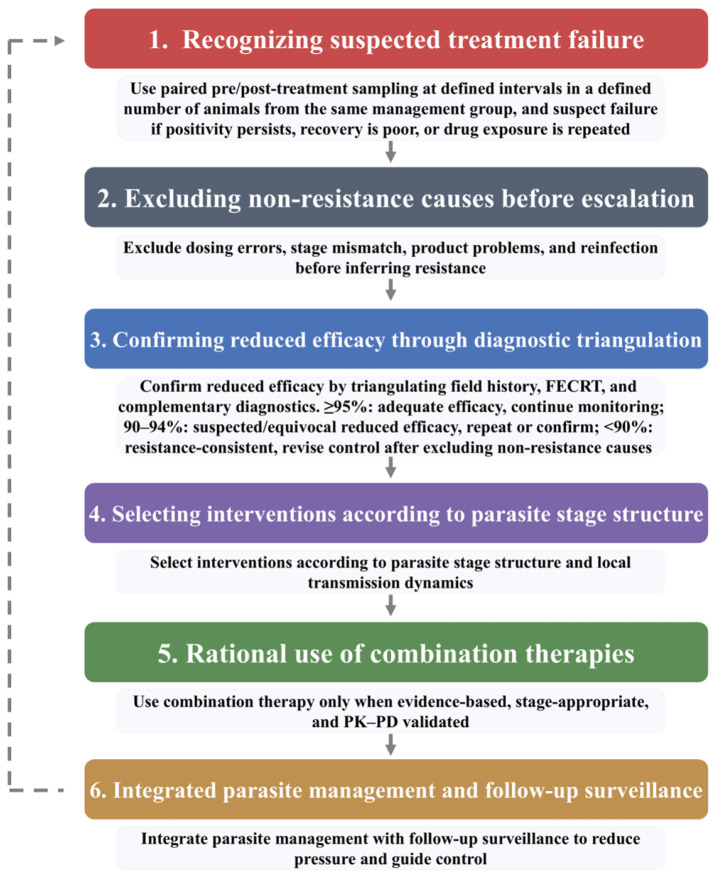
Practical on-farm diagnostic and management pathway for suspected fasciolicide treatment failure in *Fasciola hepatica*.

The management strategy proposed here is based on a closed-loop concept rather than a one-time response to apparent treatment failure. In this context, a closed-loop approach refers to a cyclical process in which suspected reduced efficacy is first recognized, then checked against operational and diagnostic evidence, followed by stage-appropriate intervention and integrated parasite management, and finally reassessed through follow-up surveillance to inform subsequent decisions (Figure 2). The purpose of this loop is not simply to change drugs after poor response, but to reduce false attribution of failure to resistance, avoid unnecessary retreatment, and limit further selection for resistant *F. hepatica* populations.

### 6.1. Recognizing Suspected Treatment Failure

The first step in management is to identify situations in which reduced efficacy should be suspected. Suspicion may arise when egg shedding persists after treatment, coproantigen remains positive, expected clinical or production recovery is not observed, or repeated use of the same fasciolicide has occurred in high-risk transmission settings. At this stage, apparent treatment failure should be interpreted as a trigger for investigation rather than immediate proof of resistance.

### 6.2. Excluding Non-Resistance Causes Before Escalation

Before resistance is inferred, non-resistance causes of poor response should be systematically excluded [104]. First, confirm correct administration, including dose rate, bodyweight estimation, dosing gun calibration, product storage. In addition, the timing of treatment should be reviewed in relation to likely parasite stage structure, because treatment given when immature flukes predominate may produce an apparent lack of efficacy if only adulticidal compounds are used. Reinfection pressure should also be considered, particularly where animals remain exposed to snail habitats shortly after treatment.

### 6.3. Confirming Reduced Efficacy Through Diagnostic Triangulation

Resistance confirmation should not rely on a single isolated assay. Instead, reduced efficacy should be evaluated through diagnostic triangulation by combining clinical or field history with FECRT and at least one complementary tool, such as CRT, and CET where feasible [60]. In practice, this means pairing appropriately timed FECRT with coproantigen testing to verify clearance, distinguish true reduced efficacy from reinfection or stage mismatch, and minimise unnecessary repeat dosing that intensifies selection pressure. Persistence of active infection should then be verified through coproantigen reduction or repeated testing at an appropriate interval before switching drug class or escalating control measures is considered. This layered approach is especially important because FECRT alone may be influenced by low baseline egg output, aggregation of parasites, reinfection, or prepatent infection.

Although universally accepted Fasciola-specific field thresholds are still lacking, the available literature supports a practical operational interpretation framework. In general, adequate efficacy may be inferred when FECRT and/or CRT show a reduction of ≥95%, particularly when confidence intervals remain compatible with high efficacy and post-treatment coproantigen testing becomes negative within the expected window [23,42,59]. Results in the range of 90–94% should be interpreted as suspected reduced efficacy or equivocal, especially if clinical response is incomplete or FECRT and CRT are discordant [39]. By contrast, reductions of <90% may be treated as operationally consistent with resistance, but only after incorrect dosing, stage mismatch, reinfection, and other non-resistance causes have been excluded, and preferably when supported by concordant CRT or CET evidence [23]. In this way, thresholds are used as decision-support tools within a triangulated framework rather than as isolated numerical cut-offs.

### 6.4. Selecting Interventions According to Parasite Stage Structure

Once reduced efficacy is suspected or confirmed, management decisions should be guided by parasite stage structure rather than by automatic drug substitution. Because most non-TCBZ fasciolicides are predominantly adulticidal, alternatives should be framed as stage-specific tools rather than simple substitutes [105]. If immature infections are expected, switching to an adulticide alone may create the appearance of poor efficacy and prompt further retreatment. Therefore, drug choice should be matched to local transmission patterns and the likely age structure of infection. In herds where TCBZ efficacy is declining, a conservative and evidence-based strategy is preferable to escalation in the absence of confirmation.

### 6.5. Rational Use of Combination Therapies

Combination therapy may represent a useful resistance management option in selected settings, particularly where compounds differ in mode of action and stage coverage. The underlying rationale is to pair agents with distinct modes of action and complementary stage activity, thereby increasing the likelihood that parasites surviving one compound are eliminated by the partner drug [36]. Field and experimental evidence indicates that some regimens can improve efficacy relative to poor-performing single-agent treatments. More recent PK–PD observations further support the view that some combinations can outperform monotherapy through altered systemic exposure as well as pharmacodynamic additivity [106]. However, combinations should not be used empirically as indiscriminate cocktails. Experimental evidence indicates that interactions can be dose- and stage-dependent; in one study, TCBZ combined with artemisinin derivatives showed synergy under some conditions but antagonism under others [107]. Accordingly, without pharmacokinetic validation and stage-specific optimisation, incorrect mixtures may reduce effective parasite exposure and inadvertently accelerate, rather than mitigate, resistance selection. Therefore, combination therapy should be considered a potentially valuable but carefully validated option, rather than a universal empirical response to suspected resistance.

### 6.6. Integrated Parasite Management and Follow-Up Surveillance

Integrated parasite management is the essential non-pharmacological backbone that sustains fasciolicide efficacy and slows resistance selection [108]. Core measures include pasture and water management to limit contact with snail habitats, grazing segregation during peak-risk periods, targeted treatment of high-risk groups rather than whole-herd dosing, and meticulous treatment records linking drug use, timing, and outcomes. These measures reduce transmission pressure and help limit repeated drug exposure. Follow-up surveillance should then be incorporated as the final step of the loop. At the regional level, harmonised surveillance should prioritise comparable phenotyping through standardised sampling intervals, transparent thresholds, and reporting of confidence intervals. In under-sampled or resource-limited settings, particularly in parts of Asia and Africa, the proposed management strategy should be adapted to local diagnostic capacity and infrastructure. Where CRT, CET, or broader laboratory support are not available, management may need to rely more heavily on repeated post-treatment monitoring, treatment history, reinfection-risk assessment, minimum operational metadata, and retention of samples for later confirmation where possible. Under these conditions, integrated parasite management may be even more important than repeated switching among limited drug options. In this way, surveillance becomes not a separate activity, but the mechanism by which one round of management informs the next.

### 6.7. Summary of the Protocol

Taken together, this management protocol provides a practical interpretation of sustainable fasciolosis control under conditions of emerging drug resistance. Rather than treating every apparent failure as confirmed resistance, the protocol emphasizes early recognition, exclusion of operational causes, confirmation through multiple lines of evidence, stage-appropriate intervention, rational combination use, and integrated surveillance. In this review, these steps are linked through a closed-loop process in which one round of assessment, intervention, and follow-up directly informs the next cycle of management. By organizing management in this way, the protocol makes currently available tools more actionable for professionals and producers while reducing avoidable selection pressure over time.

## 7. Future Directions and Research Gaps

Sustaining fasciolosis control in the face of emerging drug resistance will require progress on three key fronts, including standardized phenotyping, actionable surveillance, and mechanism-to-tool translation. First, resistance detection remains constrained by heterogeneous protocols. Priority should be given to harmonizing FECRT and CRT with agreed sampling windows, analytical thresholds, confidence intervals, and reporting checklists, and to explicitly incorporating infection intensity and stage structure into interpretation when egg output is low or prepatent infections dominate. Second, surveillance must expand beyond well-sampled settings. Coordinated, regionally representative monitoring—especially across under-sampled areas in Asia and Africa—should pair phenotypes with minimum operational metadata and include biobanking of eggs and faeces to enable retrospective validation and future genomic work. Third, moving from association to causation is essential for deployable diagnostics. The candidate mechanisms highlighted here include altered drug transport and efflux, metabolic reprogramming, stress response capacity, and drug sequestration coupled with extracellular vesicle–mediated export. These should be functionally interrogated using integrated approaches. In parallel, the field needs PK–PD-aware evaluation of formulations and combination regimens to avoid inadvertent antagonism and to identify scalable, stage-appropriate strategies. In addition, recent review evidence indicates that some natural plant products have shown in vivo efficacy against *F. hepatica*, supporting the view that plant-derived compounds may represent a complementary source of future fasciolicidal leads, even though most remain far from field-ready application [91]. Beyond its veterinary and production impact, fasciolosis also remains a zoonotic concern, and this has implications for how resistance should be viewed in the longer term. Reduced efficacy of fasciolicides in livestock may contribute to sustained environmental contamination and continued transmission risk, particularly in endemic rural settings where animal, environmental, and human interfaces are closely linked. From this perspective, improved surveillance, field-deployable diagnostics, and integrated control strategies are relevant not only for preserving drug utility in ruminants, but also for supporting broader One Health efforts to reduce zoonotic exposure. Finally, investment in field-deployable testing and integrated control will be critical to reduce reinfection pressure and slow further selection.

## 8. Review Limitations

This review has several limitations. First, the currently available evidence base remains geographically uneven, with confirmed or suspected resistance reports concentrated in Europe, Oceania, and parts of South America, whereas large areas of Asia and Africa remain under-sampled. As a result, the apparent global distribution of fasciolicide resistance may partly reflect differences in surveillance intensity and reporting rather than the true extent of the problem. Second, the available literature is subject to potential bias, including publication bias, regional imbalance in monitoring effort, and variation in study design, all of which may influence the interpretation of resistance patterns. Third, direct comparisons across studies remain constrained by methodological heterogeneity, including differences in sampling windows, baseline infection levels, parasite stage structure, and the analytical thresholds applied in FECRT- and CRT-based assessments. Finally, mechanistic understanding remains incomplete, particularly outside triclabendazole, and the absence of widely validated molecular markers continues to limit more accurate and standardised diagnosis of resistance. These limitations should be taken into account when interpreting the current resistance landscape and the practical recommendations proposed in this review.

## 9. Conclusions

In conclusion, TCBZ resistance in *F*. *hepatica* represents the most immediate threat to sustainable fasciolosis control in ruminants, largely because TCBZ is uniquely effective against both immature and adult stages and has therefore been relied upon for decades. Rather than reflex drug substitution, a practical way forward is the closed-loop management strategy outlined in this review, in which suspected treatment failure is recognised early, checked through operational and diagnostic evidence, addressed through stage-appropriate interventions integrated with parasite management measures, and reassessed through follow-up surveillance. Such an approach may help reduce unnecessary retreatment, improve interpretation of apparent drug failure, and slow further selection of resistant parasite populations. Future research should now prioritise harmonised phenotypic methods and interpretive thresholds, broader surveillance in under-sampled regions, and the translation of candidate resistance mechanisms into deployable diagnostic tools. Progress in these areas, together with PK–PD-aware evaluation of formulations and combination regimens, will be essential for preserving remaining drug utility and improving the long-term sustainability of fasciolosis control.

## Figures and Tables

**Table 1:** Major anthelmintic drugs used for fasciolosis in ruminants.

Group	Drug	Primary Stage(s) Targeted	First Reported Veterinary Use	First Report of Reduced Efficacy or Resistance	Administration Routes
Benzimidazoles	Triclabendazole	Both adults and immature	First reported in 1983	First confirmed in Australian sheep in 1995	Oral, pour-on
	Albendazole	Adults	First reported in 1976	First reported in Spanish sheep in 2006	Oral, intraruminal
Salicylanilides	Closantel	Adults and late immature	First reported in 1977	First reported of treatment failure in Sweden beef cattle in 2014	Oral, pour-on, injectable
	Oxyclozanide	Adults	First reported in 1966	Not reported	Oral
	Rafoxanide	Adults	First reported in 1970	First evidence of resistance in Egypt cattle in 2013	Oral
Sulphonamide	Clorsulon	Adults	First reported in 1977	First evidence of resistance in Spanish sheep in 2014	Injectable
Halogenated phenol	Nitroxynil	Adults	First reported in 1969	Not reported	Injectable

**Table 2:** Methods for detecting and confirming fasciolicide resistance in *Fasciola hepatica*.

Method		Core Readout	Use in Monitoring Workflow	Main Strengths	Main Limitations
FECRT	Conventional FECRT	Pre/post FEC reduction	Screening for reduced efficacy	Low-cost; field-feasible	Unstable when baseline FEC is low; sensitive to sampling error and egg-shedding dynamics; cannot separate resistance from reinfection, stage mismatch and operational failure
	FECPAKG2/ParaSight	Digitised egg detection	Operational extension of egg-based monitoring	Improves standardisation for field workflows	Cost and limited validation for resistance workflows; inherits egg-based confounders
	FLOTAC	High-recovery copromicroscopy	Enhanced egg detection to support efficacy testing	Higher sensitivity/precision than traditional methods; suited to pooled testing	Inherits egg-based confounders
CRT (cELISA)		Coproantigen reduction	Independent confirmation alongside FECRT	Detects active infection; sensitive at low burdens; useful when FEC is low and prepatent	Timing-dependent; lacks genotyping capability for resistance
CET		Necropsy-based fluke survival	Definitive confirmation	Direct survival endpoint; immature and adult stratification; validates FECRT and CRT	Resource- and ethics-intensive; not scalable for routine surveillance
EDHT		In vitro egg hatching under drug	Adjunct phenotyping	Cost-effective lab phenotyping; can use eggs from single animal	Protocol heterogeneity limits comparability and thresholds
Serology		Antibody responses	Surveillance mapping	Herd-level risk stratification; exploratory biomarker signals	Antibody persistence limits post-treatment inference; not suited for clearance confirmation
Molecular diagnostics	PCR/qPCR	Parasite DNA detection	Supportive diagnosis	Higher throughput; species differentiation	Sensitivity depends on eggs concentration; no validated resistance loci
	LAMP/RPA	Isothermal amplification signal	Field-deployable detection adjunct	High analytical sensitivity; suited for field testing	Sample preparation is critical; no validated resistance loci

**Table 3:** Mechanistic hypotheses and evidence strength for fasciolicide resistance in *Fasciola hepatica*.

Group	Drug	Main Mechanism of Resistance	Key Uncertainties	Validated Markers
Benzimidazoles	Triclabendazole	Reduced drug exposure and associated tolerance pathways	Primary causal mechanism; relative contribution of each pathway; reproducibility across isolates and populations	No routine validated marker; major resistance locus identified
	Albendazole	β-tubulin-related target biology without a simple target-site model	Whether reduced susceptibility is target-site, regulatory, or broader class-level	No validated marker
Salicylanilides	Closantel	Altered energy metabolism/oxidative phosphorylation uncoupling	Drug-specific resistance mechanisms and their consistency across compounds	No validated marker
	Oxyclozanide			
	Rafoxanide			
Sulphonamide	Clorsulon	Mechanism currently undefined	Whether reduced efficacy reflects drug-specific resistance or broader multidrug selection	No validated marker
Halogenated phenol	Nitroxynil	Oxidative phosphorylation uncoupling with possible membrane effects	Both mode of action and resistance basis remain insufficiently characterized	No validated marker

## Data Availability

No new date were analyzed or created during this study. Data sharing is not applicable.

## Abbreviations

The following abbreviations are used in this manuscript:

ABZ	Albendazole
ABC	ATP-binding cassette
CET	Controlled efficacy test
cELISA	Coproantigen enzyme-linked immunosorbent assay
CRT	Coproantigen reduction teste
EDHT	Egg development and hatching test
FABP	Fatty acid-binding protein
FEC	Faecal egg count
FECRT	Faecal egg count reduction test
FMO	Flavin-containing monooxygenase
GST	Glutathione S-transferase
LAMP	Loop-mediated isothermal amplification
PCR	Polymerase chain reaction
qPCR	Quantitative polymerase chain reaction
RPA	Recombinase polymerase amplification
TCBZ	Triclabendazole
TCBZ-R	Triclabendazole-resistant
TCBZ-S	Triclabendazole-susceptible
TCBZ-SO	Triclabendazole sulfoxide
TCBZ-SO_2_	Triclabendazole sulfone
